# The Effectiveness of Fluorescent Light Energy as Adjunct Therapy in Canine Deep Pyoderma: A Randomized Clinical Trial

**DOI:** 10.1155/2021/6643416

**Published:** 2021-01-09

**Authors:** Andrea Marchegiani, Alessandro Fruganti, Andrea Spaterna, Matteo Cerquetella, Adolfo M. Tambella, Susan Paterson

**Affiliations:** ^1^School of Biosciences and Veterinary Medicine, University of Camerino, Camerino, Italy; ^2^Virtual Vet Derms Ltd. Lakeview, 3 High Birkrigg Park, Stainton, Kendal LA80DY, UK

## Abstract

A single centre, single-blinded, prospective, randomized, controlled clinical study was conducted to evaluate the effectiveness of twice weekly fluorescent light energy therapy (Phovia™) as adjunct to systemic antibiotics in the management of deep pyoderma in dogs. Dogs with clinical lesions consistent with deep pyoderma, positive bacterial culture, and showing neutrophil engulfing bacteria at cytology were included in the study. Assessments were undertaken weekly for 8 weeks and every 2 weeks thereafter until 12 weeks after enrolment. At each visit, lesions were scored and cytology was conducted to determine a neutrophil engulfing bacteria score. All dogs (Groups A and B) were treated with systemic antibiotic twice daily, and Group B received additionally Phovia twice weekly. Median treatment duration was 11.7 weeks for Group A and 5.7 weeks for Group B. After 8 weeks of treatment, the percentage of dogs that achieved clinical resolution was 35.0% and 88.0% for Groups A and B, respectively. Lesion scores showed highly statistically significant difference in favour of Group B from week 3 to 8, and neutrophil engulfing bacteria scores showed statistical difference from week 2 onwards in favour of Group B. These results indicate that Phovia, when used as an adjunct to systemic antibiotics, can accelerate time to clinical resolution in cases of canine deep pyoderma.

## 1. Introduction

Bacterial skin infection or pyoderma is a common canine dermatological problem [1]. Pyoderma can be defined in a number of ways, and the most common of which is by depth of infection. This can be surface, superficial, or deep infection, of which each can be either localised or generalised. Canine deep pyoderma (CDP) is defined as infection that extends beyond the dermoepidermal junction into the dermis and panniculus and/or infection that progresses through follicular damage from a folliculitis to involve the deeper tissues [1–3]. Histopathologically CDP produces a deep suppurative process with accompanying pyogranulomatous folliculitis, furunculosis, cellulitis, and panniculitis [4–6]. Although CDP can be an idiopathic disease, it is more commonly associated with an underlying cause, such as allergy (atopic dermatitis and cutaneous adverse food reaction), ectoparasites (*Demodex canis*), and endocrine disease (hypothyroidism and hyperadrenocorticism) [7]. Although topical therapy can be used successfully as monotherapy in cases of surface and superficial pyoderma [8], it is more frequently used in combination with systemic antibiotic therapy in cases of deep infection [6]. Current recommendations suggest cases of CDP should be treated with systemic antibiotics at the upper end of their dose rate, for a minimum of 4–6 weeks together with topical antiseptic therapy [6]. Treatment should be continued until the infection has resolved visually and cytologically [6, 9]. Antibiotic courses in practice are often much shorter than 4–6 weeks due to a lack of owner compliance and financial constraints, especially where large dogs are treated. Where follicular damage occurs as a sequel to infection, keratin and hair can be released into the dermis leading to a foreign body reaction and the persistence of clinical signs that may warrant anti-inflammatory therapy after the infection has resolved [10].

Low-energy light therapy, also known as photobiomodulation (PBM), has been shown to have beneficial effects in several skin conditions in animals as well as anti-inflammatory properties [11, 12]. A Fluorescent Light Energy (FLE) system that consists of a blue light-emitting diode (LED) device and a topical photoconverter gel, which when illuminated by the LED device, emits low-energy light in the form of fluorescence has shown beneficial effects in management of dermatological conditions and chronic wounds in humans [13, 14]. A specific FLE system has been developed for animal use (Phovia™, Klox Technologies Limited, Ireland) which has been successfully applied in the management of different conditions including, interdigital pyoderma, otitis, wounds, and canine perianal fistulae [15–19].

The aim of this study was to assess whether the adjunction of Phovia with systemic antibiotics could lead to a reduction in time to clinical healing, measured as the percentage of dogs that reached clinical resolution by 8 weeks, compared to dogs which received antibiotics alone.

## 2. Materials and Methods

This study was designed as a prospective, single-blinded, randomized study and conducted in Veterinary Teaching Hospital of Camerino University, Italy. Thirty-five dogs affected by CDP and presenting with a range of clinical lesions, including crusted papules, haemorrhagic vesicles and bullae, haemorrhagic crusts, ulcers, erosions, and fistulae with draining tracts, were enrolled in the study (Table 1).

Dogs were randomly assigned to either Group A (systemic antibiotic, 18 dogs) or B (systemic antibiotic plus Phovia, 17 dogs) using iMedNet software (iMedNet Solutions, Minnetonka MN USA), based on a randomization list created using SAS Software version 9.4. The principal investigator (PI) was the one that scored the dogs on the day of enrolment and then weekly. The collaborating investigator (CI) conducted randomization at enrolment, treatment visits, and all other scheduled assessments. The PI remained blinded on group allocation and treatment received until the end of the study; weekly lesion photographs were uploaded by CI to iMedNet cloud-based software for scoring by the blinded PI. The study protocol was in compliance with European legislation on the protection of animals used for scientific purposes and approved by the University of Camerino Ethics Committee (Prot. N. 1/2017); in addition, written informed owner consent was granted in all cases.

### 2.1. Diagnostic Tests

Skin scrapings and cytology (impression smears and tape strippings) were performed before enrolment on all dogs to check for ectoparasites and *Malassezia* spp. infection. Samples were also taken for culture and susceptibility from all dogs at the time of enrolment. These were obtained from fistulae with draining tracts, ulcers, or erosions and from the underside of crusts or papules, if present. In addition, routine haematology and biochemistry together with endocrine function tests were performed to exclude dogs with signs of systemic ill health, hyperadrenocorticism, hypothyroidism, and diabetes mellitus. Fungal cultures were performed to rule out dermatophytosis, and serology was taken to ensure no dogs were suffering with leishmaniasis. Dogs were placed on an exclusion diet throughout the trial period to rule out cutaneous adverse food, and good flea control was maintained. Feeding and housing conditions were kept unchanged during the length of the study.

### 2.2. Exclusion Criteria

Dogs were excluded where rods or mixed infection with rods and cocci were identified on cytology or where a multiresistant bacterium was isolated on subsequent culture and susceptibility testing. They were also excluded if *Demodex canis* or *Malassezia* spp. was identified on skin scrapings or cytology. Animals younger than 12 months, breeding animals or pregnant or lactating females were also excluded. Any dog that had had treatment with systemic antibiotics, antihistamines, glucocorticoids, ciclosporin, or topical anti-inflammatory or antimicrobial therapy for two weeks prior to enrolment in the trial were excluded. Lokivetmab was unavailable as a treatment option, and no dogs were on oclacitinib at the time of enrolment. Concurrent therapy with any of these therapies was not permitted throughout the course of the trial. Dogs affected by interdigital pyoderma were not considered for the scope of the present study, being already explored in a previous investigation [15]. Number of CDP episodes did not represent an exclusion nor inclusion criterion.

### 2.3. Inclusion Criteria and Scoring System

In view of the lack of validated scoring systems for CDP, investigators developed an empirical scoring chart based on a 0–4 score of four lesion types (Table 1) resulting in a global lesion score (GLS) range of 0–16. Dogs were scored at enrolment and weekly to monitor lesion evolution over time.

For inclusion in the study, dogs had to score 3 or 4 in at least one of these four clinical parameters. Neutrophil engulfing bacteria score (NES) ranging 0–4 (Table 2) was obtained by pressing a clean microscope slide directly onto all lesions and staining with a Romanosky (Diff-Quik) stain. For a dog to be enrolled, its NES score had to be at least 1.

At enrolment, coat around CDP areas was clipped and this procedure was repeated throughout the study if necessary. All dogs were prescribed oral cephalexin therapy (20 mg/kg p.o. q 12 h), and a swab sample was taken for culture and susceptibility. Samples were taken from fistulae with draining tracts, ulcers or erosions, and under crusts, if present, and immediately sent to the laboratory for bacteria isolation and identification using standard techniques; susceptibility testing was performed using diffusion disk technique. Results were provided within 5 days and if the identified bacteria were not susceptible to cephalexin, another suitable antibiotic would have been chosen instead of cephalexin and the dog withdrawn from the trial. For all dogs, antibiotic therapy was continued until 7 days after clinical resolution determined by visual and cytological resolution of lesions (GLS scored 0). Dogs in Group B received, in addition to the systemic antibiotic therapy, Phovia twice weekly until clinical resolution was determined. As previously described (15), the Phovia procedure consisted of applying an approximate 2 mm layer of the gel in the lesions and illuminating with the blue light-emitting diode (LED) device that delivers noncoherent blue light with peak wavelength between 440 and 460 nm and a power density between 55 and 129 mW/cm^2^, for 2 minutes, at approximately 5 cm distance. After illumination, the gel was gently removed using sterile gauzes dipped in sterile saline solution. Dogs were kept by their owner during Phovia application, and no sedation or excessive contention was needed. If crusts were present, they were not removed prior to Phovia application. Since the goal of the present study was to assess a possible beneficial effect of Phovia adjunction to systemic antibiotics in CDP cases, topical antimicrobial shampoos were not permitted in neither group. Feeding and housing conditions were maintained on a consistent basis during the length of the study.

Assessments were performed at enrolment (day 0) and then on a weekly basis until week 8 for subjects that reached clinical resolution at day 56 or before. For those dogs not reaching clinical resolution by day 56 (week 8), further assessments were conducted on days 70 and 84 until clinical resolution was determined. Dogs that did not reach clinical resolution by week 12 continued treatment until clinical resolution. For a decision of PI and CI, C&S testing would not be repeated in these dogs if global lesion scores showed a constant improvement with weeks.

The percentage of dogs that reached clinical resolution by week eight (day 56 assessment) was the primary efficacy endpoint. All dogs achieving clinical resolution at any time up to a maximum of 12 weeks of treatment were enrolled for a further 4-month follow-up period to assess for any recurrence of lesions, defined as the reappearance of one or a combination of lesions as per Table 1 in the same previously affected site. Dogs not achieving clinical resolution by 12 weeks were not enrolled in the follow-up study. During the follow-up period, owners phoned monthly to determine if they noticed any signs of relapse or if the dog had required any antibiotic or anti-inflammatory therapy for the CDP or other condition. All owners were invited for a final clinical examination four months after the start of the follow-up period.

All data analyses (Student's *t*-test, Fisher exact test, and Mann–Whitney tests) were conducted using SAS v9.4 considering significant values of *p* ≤ 0.05.

## 3. Results

**Figure 1:**
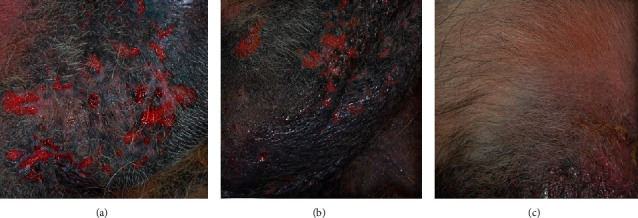
Group B case #8 clinical evaluation visit pictures. (a) Enrolment visit; (b) day 21 visit; (c) day 42 visit; clinical resolution was determined.

Thirty-five dogs were enrolled, 18 were randomized in Group A (systemic antibiotic), and 17 in Group B (systemic antibiotic plus Phovia, Figure 1).

During the study, 1 dog from Group A was withdrawn at week 6 due to poor owner compliance. No significant differences in sex distribution, age, body weight, and number of purebred dogs were identified (Table 3). Dietary regimen did not influence the severity of the lesions.

Similarly, no differences were also found between groups in terms of severity of condition at enrolment (T-tested): Group A had an average GLS of 8.59 ± 1.91sd; Group B 8.65 ± 1.54sd. There was only one isolated, mild vomiting episode in a dog from Group B which resolved without any additional treatment and did not require any change of treatment protocol.

Predominant pathogen isolated was *Staphylococcus* spp. (*n* = 35); isolated species were *S. pseudintermedius*, *S. aureus*, and *S. xylosus*. A number of other different bacteria (*Streptococcus* spp., *E. coli*, and others) were detected unfrequently.

**Figure 2:**
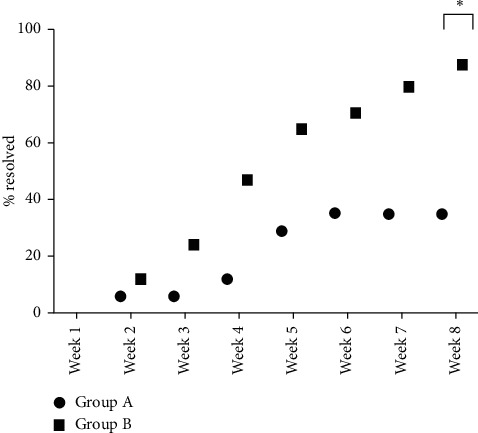
Percentage of dogs healed by treatment and study week. ^∗^*p* < 0.01.

Group B showed a statistically significant improvement in the percentage of dogs achieving clinical resolution at week 8, as shown in Figure 2.

By week 8, this percentage was 35% and 88% for Groups A and B, respectively. This is a highly statistically significant difference in favour of Group B treated patients (*p* < 0.01).

In Group A, the mean time to achieve clinical resolution was 11.7 ± 6.3 weeks (median 12.0 weeks), while in Group B, it was 5.7 ± 3.5 weeks (median 5.0 weeks).

**Figure 3:**
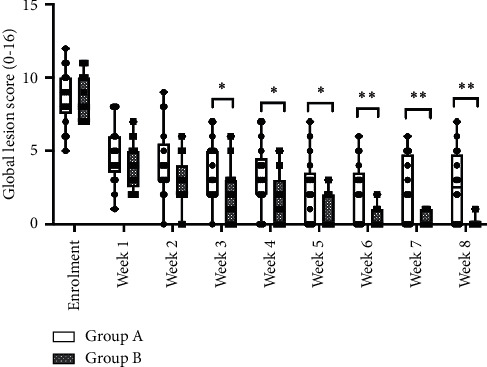
Average global lesion scores by treatment and study week. ^∗^*p* < 0.05; ^∗∗^*p* < 0.01.

Starting from week 3, a statistically significant decrease of GLS in favour of Group B was observed (Figure 3).

**Figure 4:**
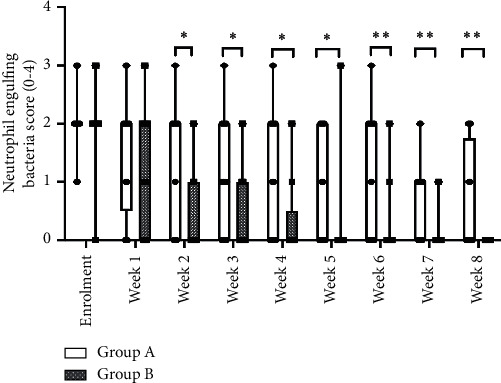
Average neutrophil engulfing bacteria scores by treatment and study week. ^∗^*p* < 0.05; ^∗∗^*p* < 0.01.

At week 8, the average GLS was 2.50 for Group A and 0.12 for Group B (*p* < 0.001). NES scores registered a statistically significant improvement from weeks 2 onwards in favour of Group B, as shown in Figure 4.

Four dogs from Group A and 16 from Group B were enrolled for the follow-up assessment period. The reason why there were fewer dogs from Group A enrolled is because more dogs in this group did not achieve clinical resolution by week 12, which was one of the requirements to enrol in the follow-up period. No recurrence of deep pyoderma was recorded in either group during the 4-month follow-up period.

## 4. Discussion

This study has shown that Phovia is well tolerated by patients when used in the treatment of CDP, and no adverse effects were recorded in any case where it was used as part of the treatment protocol. It significantly shortens the time to clinical resolution of CDP when used as an adjunct to systemic antibiotics, accelerating the time to clinical resolution and consequently reducing the duration of systemic antibiotic treatment. A previous study from Scapagnini and collaborators shed light on the mode of action of FLE [21]. This study found that Phovia-exposed skin exhibits less inflammation and complete reepithelialization due to a significant downregulation of the expression of TNF-*α* and upregulation of EGF, FGFs, TGF-*β*, Coll I and III, Ki67, FVIII, and DCN. In the same study, Phovia was shown to modulate mitochondria biogenesis with a substantial increase (close to 90%) in the number of mitochondria from baseline: as a result, the amount of adenosine triphosphate is augmented at a cellular level, further supporting the healing process [21]. Phovia has been previously applied for the management of interdigital furunculosis in dogs, using a similar intervention as for the present study and obtaining superimposable results, with a percentage of dogs that achieved clinical resolution by week 6 of 26.5% and 84.6% for control and Phovia groups, respectively [15]. In this study, in the group where antibiotics were used as the sole form of therapy (Group A), resolution was achieved in a mean time of 11.7 weeks whereas the group treated with antibiotics and Phovia (Group B) resolved in a mean time of 5.7 weeks. Group B also showed a statistically significant difference in speed of resolution of the infection at 8 weeks. Cases of CDP can often show signs of improvement within 2–3 weeks, but a complete resolution of clinical signs can take 4–6 weeks or even longer [6, 22]. Based on previous studies that have considered treatment durations required to achieve clinical resolution, it occurred in an average healing time between 4 and 8 weeks, even if longer periods may be required [4, 9, 23]. Considering the dogs from Group A of the present study, only 35% reached clinical resolution at week 8; in aforementioned studies, severity of CDP was not evaluated and it could be supposed that dogs from the present study had more severe disease than those of previous ones, even if this can hardly be confirmed. Any therapy should be continued until clinical signs have resolved and cytology is normal. In cases of deep pyoderma, current recommendations are that antibiotic therapy should continue for 14 days after the resolution of clinical signs [5, 24]. However, there is little evidence to support these recommendations which are mostly anecdotal. In addition, the efficacy of medication can be compromised, and the development of resistance created, when long courses of antibiotics are prescribed. This may be due to poor levels of owner compliance due to under dosing, missing doses or stopping treatment too early because the clinical signs have resolved [25, 26]. Topical therapy management may be a useful adjunct to systemic antibiotics, but there are insufficient studies to support a reliable recommendation as sole treatment for deep pyodermas [5, 22]. In the present study, all owners (except for the one who withdrawn his dog) were compliant with the study schedule of bi-weekly visit. Topical antimicrobial therapy was not an option, and it would be desirable to have further investigations including also this management modality, especially for those dogs treated only with systemic antibiotic. Case selection in this study relied on the identification of cases of CDP which is poorly defined in the literature. The diagnosis of pyoderma was based on the characteristic skin lesions and the cytological evidence of intracellular bacteria [3, 27, 28]. Phagocytosed bacteria within the cytoplasm of neutrophils is recognised as a useful indicator of the presence of infection rather than absolute bacterial numbers, as extracellular bacteria seen in section can be contaminants [15, 29, 30]. Pruritus was not reported as a consistent finding by owners, so this was not included as a means of recording response to therapy; however, retrospectively this may have been a useful inclusion. Despite the lack of a recognised lesion scoring system for deep pyoderma, no significant differences were seen at enrolment between patients of the two treatment groups using the scoring system proposed by the authors; therefore, changes in scores between the two groups with the progression of the study were deemed to be an indication of the differing responses to therapy. Deep pyodermas are multifactorial and usually secondary to conditions such as endocrinopathies, ectoparasites, metabolic disturbances, or other underlying disease; recurrence is not infrequent unless such primary diseases are properly addressed, requiring periodic therapy and management [6, 26, 31]. In these cases, it is likely that pyoderma was either idiopathic or due to canine atopic dermatitis as all other causes were investigated and eliminated. In future studies, specific therapy for underlying atopic dermatitis may be useful to prevent recurrence once the infection has resolved. The perpetuating tendency of CDP is often frustrating for pet owners and can compromise general wellbeing and potentially interactions between affected dog and owners, thus negatively affecting the QoL of dogs and their owners [32–35]. A limitation of the present study was the lack of pruritus evaluation in CDP cases. A further improvement in this study could have been the addition of quality of life (QoL) scoring for both the pet and the owner throughout the trial period.

## 5. Conclusions

Phovia has already shown potential to be a useful tool in the management of interdigital pyoderma, otitis, wounds, and perianal fistulae in dogs [15–19]. This study suggests that Phovia is a useful tool also in the management of CDP when used as an adjunct therapy with systemic antibiotics, improving resolution of the infective process, increasing the level of owner compliance, and accelerating the healing of the lesions.

## Figures and Tables

**Table 1:** Scoring system for CDP.

Lesion type		Haemorrhagic vesicles/bullae	Fistula with draining tracts	Haemorrhagic crust/papules	Ulcers/erosions
Score	Grade				
0	Healed	Absent	Absent	Absent	Absent
1	Mild	Less than 2 lesions per 100 cm^2^	Less than 2 lesions per 100 cm^2^	Less than 2 lesions per 100 cm^2^	Less than 2 lesions per 100 cm^2^
2	Moderate	3–5 lesions per 100 cm^2^	3–5 lesions per 100 cm^2^	3–5 lesions per 100 cm^2^	3–5 lesions per 100 cm^2^
3	Severe	6–10 lesions per 100 cm^2^	6–10 lesions per 100 cm^2^	6–10 lesions per 100 cm^2^	6–10 lesions per 100 cm^2^
4	Very severe	More than 11 lesions per 100 cm^2^	More than 11 lesions per 100 cm^2^	More than 11 lesions per 100 cm^2^	More than 11 lesions per 100 cm^2^

**Table 2:** Severity of neutrophil engulfing bacteria scores (NESs).

NES score	Numbers of neutrophils engulfing bacteria^∗^
0	None seen
1	<1
2	1–4
3	5–10
4	>10

^∗^Numbers of neutrophil engulfing bacteria per high powered field were counted at x500 magnification and an average was taken over 10 microscopic fields.

**Table 3:** Summary table for each dog enrolled in the study.

Allocation	#	Age (years)	Breed	Sex	Weight (kg)	BCS	Coat type	Global lesion score at enrolment	NES score at enrolment	CDP localization	CDP episode prior to enrolment	Number of Phovia applications for healing	Number of sites treated with Phovia
Group A	1	8	Mixed breed	m	42	4	L	9	3	Flank	Yes		
	2	12	GSD	m	20	4	L	6	2	Dorsum	No		
	3	2	Pointer	m	21	4	S	8	2	Flank	No		
	4	11	GSD	m	40	6	L	8	1	Flank	No		
	5	16	Mixed breed	f	26	6	L	5	2	Abdomen	Yes		
	6	5	GSD	f	25	4	L	6	2	Thigh	Yes		
	7	3	Mixed breed	m	36	5	S	9	2	Dorsum	Yes		
	8	12	Labrador	f	37	6	L	11	2	Abdomen	No		
	9	10	Mixed breed	f	24	4	S	10	1	Back	Yes		
	10	6	GSD	m	22	5	S	12	2	Flank	Yes		
	11	14	Mixed breed	m	32	5	L	8	2	Dorsum	No		
	12	14	Mixed breed	m	25	5	S	9	2	Thigh	No		
	13	3	Mixed breed	m	40	6	S	8	2	Dorsum	Yes		
	14	8	Boxer	m	30	6	S	7	1	Abdomen	No		
	15	5	Dobermann	m	38	7	S	9	2	Thigh	No		
	16	13	Mixed breed	f	22	6	S	11	3	Back	Yes		
	17	11	Mixed breed	m	32	6	S	10	2	Flank	Yes		
	18^∗^	8	GSD	m	34	6	L	11	3	Abdomen	No		
				*mean*	30.12	5.2							
				*sd*	7.55	1.0							

Group B	1	16	Mixed breed	f	18	3	S	8	1	Dorsum	No	8	2
	2	7	Mixed breed	f	34	5	L	8	2	Flank	No	25	3
	3	5	Mixed breed	m	36	4	L	11	3	Abdomen	Yes	17	2
	4	2	Mixed breed	m	42	5	L	11	3	Back	No	8	1
	5	12	Mixed breed	m	40	6	L	7	3	Dorsum	No	6	2
	6	7	Bull terrier	m	23	5	S	8	2	Thigh	Yes	10	3
	7	6	Bull terrier	m	25	4	S	8	2	Dorsum	No	14	3
	8	11	GSD	f	25	5	L	9	2	Abdomen	Yes	8	1
	9	6	GSD	m	40	5	L	7	2	Flank	Yes	14	2
	10	8	Pitbull	m	25	5	S	10	2	Dorsum	No	14	1
	11	10	Bulldog	m	26	6	S	10	2	Back	Yes	12	1
	12	14	Mixed breed	f	25	4	S	10	2	Back	No	10	1
	13	1	Labrador	f	30	5	L	7	2	Flank	No	4	2
	14	5	Mixed breed	m	24	5	S	8	2	Dorsum	Yes	4	2
	15	4	Labrador	m	35	6	L	11	2	Abdomen	No	8	3
	16	4	Mixed breed	m	23	5	S	7	3	Abdomen	Yes	6	2
	17	4	German hunting dog	f	20	4	S	7	2	Flank	No	10	1
				*mean*	28.88	4.8							
				*sd*	7.47	0.8							

BCS: body condition score, evaluated according to [20]; GSD: German Shepherd Dog; sd: standard deviation. ^∗^Withdrawn at week 6 due to poor owner compliance.

## Data Availability

The data used to support the findings of this study are available from the corresponding author upon request.
